# Over-expression of c-Myc oncoprotein in oral squamous cell carcinoma in the South Indian population

**DOI:** 10.3332/ecancer.2008.128

**Published:** 2009-02-23

**Authors:** RB Pai, SB Pai, RM Lalitha, SV Kumaraswamy, N Lalitha, RN Johnston, M Bhargava

**Affiliations:** 1 Kidwai Memorial Institute of Oncology, Hosur Road, Bangalore 560029, India; 2 Georgia Institute of Technology, EST Building, 311 Ferst Drive, Atlanta, GA 30332, USA; 3 Department of Oral and Maxillofacial Surgery, M.S. Ramaiah Dental College and Hospital, Bangalore, India; 4 VS Dental College and Hospital, Bangalore 560004, India; 5 Department of Biochemistry and Molecular Biology, The University of Calgary, Calgary, Alberta, Canada T2N 4N1

## Abstract

Oral neoplasm constitutes a predominant class of cancer that is encountered in South India. This is in large part due to the elevated risk of oncogenesis as a result of the habit of chewing of quids containing betel leaves, areca nut and smokeless tobacco. An array of molecular events are induced during the transformation of the buccal epithelium, among them the over-expression of oncogene products plays a key role. The c-Myc protein, a regulator of a number of key cellular signalling pathways, plays a pivotal role in a number of malignancies. The present study was undertaken to evaluate expression of the c-Myc protein in tumours of the oral cavity from the South Indian population, predominantly oral squamous cell carcinoma (OSCC). The c-Myc protein was over-expressed in 80% of the cases studied. Taking into account the pivotal role demonstrated for c-Myc in tumourigenesis, our observations suggest a key role for Myc oncoprotein in the genesis of OSCC as well as its potential as a therapeutic target in this population.

## Introduction

In Southern India, of the various classes of malignancies presenting at the clinic, a large proportion are cancers of the oral cavity. Oral malignancies in this population are unique as these are generated by biological alterations due to insult by physical and chemical agents due to the use of quids for extended periods of time [1,2]. In experimental models, the ingredients from the quids have demonstrated induction of cellular and molecular changes, including the generation of reactive oxygen species (ROS) and DNA damage in the hamster pouch model [3,4]. In a study conducted on 140 patients, with age- and sex-matched controls, with oral cancers from Central India, tobacco use, smoking and alcohol consumption emerged as contributing factors that act in a synergistic fashion in the induction of oral neoplasia [5]. Furthermore, multi-step oral epithelial carcinogenesis models induced in the laboratory employing systematic genetic alterations clearly indicated that in addition to changes in cyclin D1 and p53, c-Myc expression along with the acquisition of telomerase activity was pivotal for neoplastic transformation of these tumours [6].

Even though the contribution of quid chewing to elevating the oral cancer risk is undisputed, the diversity in the use of the various combinations of areca, betel leaves and tobacco in various countries as well as in the various regions in India [1] warrants comprehensive molecular characterization of the oral cancers from this region. A number of studies by cancer researchers with respect to molecular mechanisms underlying oncogenesis have shown the emergence of c-Myc expression as a key event (for reviews: [7–9]). Detailed studies have shown that this is in part due to the seminal role the Myc oncoprotein plays in a number of cellular signalling pathways. Transcriptional regulation of the telomerase, a key molecule in solid tumours [10] as well as leukaemias [11], is shown to be mediated by the Ets transcription factor family with the involvement of c-Myc [12]. Similarly, the WNT1 signalling pathway also targets c-Myc during tumour progression [13], whereas the elevated prolyl isomerase, Pin1 increases the transcription of c-Myc [14]. Genome-wide profiling of OSCC has also shown that, among other genes, gain of c-Myc gene occurred at a high frequency [15]. Even though gene amplification mainly contributes to elevated c-Myc protein levels, alternate modes of regulation mediated via c-Myc specific transcription factors can also lead to a similar end point [16]. Hence, in the present investigation, c-Myc protein identification in oral cancers was made the focus of the study.

Additionally, therapeutic strategies that target c-Myc hold promise in the management of cancers that over-express the Myc oncoprotein [17].

## Patients and methods

### Patient and tumour sample

Tumour samples used in the study were from patients presenting with tumour of the oral cavity at the Regional Cancer Center-Kidwai Memorial Institute of Oncology, Bangalore, India. History of the patients included in the study indicated that they were all habitual chewers of quid with betel leaves, areca nut and tobacco and retained the quid in the mouth for prolonged periods of time. The age of the patients ranged from 32 to 80 years. The SCC were diagnosed and graded using the following criteria: grade I, well differentiated; grade II, moderately differentiated. The following parameters were used for tumour-node-metastasis (TNM) staging. For tumour size, T1: 0–2 cm; T2: 2–4 cm; T3: > 4 cm; and T4: extensive spread of the tumour, or if there is involvement of nerve or bone. N0: no clinically palpable cervical lymph nodes or palpable nodes, but metastasis suspected. N1: clinically palpable-contra/homo lateral nodes that are not fixed, metastasis suspected. N2: clinically palpable contra lateral or bilateral cervical lymph nodes that are not fixed, metastasis suspected. N3: clinically palpable lymph nodes that are fixed, metastasis suspected. M0: No distant metastasis; M1: clinical and radiographic evidence of metastasis.

### Tissue specimens and immunohistochemistry

Tumour tissue samples were obtained by biopsy as well as by surgery. The tissue specimens were fixed in 10% formalin prior to embedding in paraffin wax. Sections of the tumour were stained with Haematoxylin-Eosin (H&E) and graded for the stage of the disease by the pathologists. For immunoreactivity studies with c-Myc, 5 μM paraffin sections on glass slides were deparaffinized and processed for studies. Immunohistochemistry was performed using anti-Myc antibodies (Oncor) by standard protocols as described earlier [18]. Counterstaining was done with haematoxylin. Sections were mounted in glycerin for microscopic analysis. Treatment of tissue sections with all the reagents except the anti-Myc antibody served as controls. Analyses for the immunoreactivity of the antibodies were performed by light microscopy.

## Results

The majority of the tumours in the cancer patient population included in the present study were diagnosed as squamous cell carcinoma (Table 1). There was a preponderance of cases in the T3/T4 stages of disease. Immunohistochemical analysis revealed that 80% of the tumours expressed the c-Myc oncoprotein. A case of squamous cell carcinoma of the soft palate that was diagnosed as grade I was negative for anti-Myc staining. Among the other tumours that were negative were two cases of grade II as well as grade III OSCC. Interestingly, an epidermoid carcinoma, stage IV did not show elevated expression of the c-Myc protein. There were no detectable levels of the Myc oncoprotein in the fibrous tissue of a patient who presented with inflammation but no malignancy. Dysplastic lesions of the tongue displayed positive c-Myc immunoreactivity. This was also the case for acanthotic-stratified epithelium. One case of verrucous carcinoma, a variant of the SCC, that was investigated in the current study showed a moderate level of intensity for anti-Myc staining.

**Figure 1:**
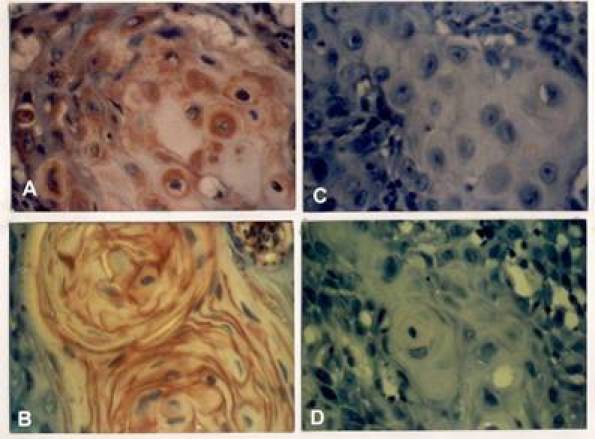
Immunohistochemical staining for Myc oncoprotein in oral squamous cell carcinoma. Tumour sections were processed as detailed in the *Methods* section. The sections were examined under light microscopy for positive staining (A and B) and lack of reaction (C). Control assays were performed in parallel with the exclusion of the anti-Myc antibody from the reaction (D).

Representative results of immunohistochemistry for anti-Myc are depicted in Figure 1. Tissue sections judged as positive showed distinct staining in the tumour [Figure 1A and B]. Tumour tissue(s) that lacked this was scored as negative for the Myc oncoprotein [Figure 1C]. Tissue sections that were processed with all the reagents except the anti-Myc antibody failed to show any reactivity, confirming the specificity of the reaction observed in the positive samples [Figure 1D].

## Discussion

Cancer of the oral cavity is a neoplasia, which occurs with high frequency in the South Indian population due to quid chewing habits. Even though the habit is largely prevalent in the Indian subcontinent, the ingredients of the quid vary from region to region [1]. We wanted to determine, within the South Indian population, the key tumour markers that are expressed in oral carcinoma that could have prognostic as well as diagnostic implications. Previously, we have reported the expression of carcinoembryonic antigen (CEA) in a number of OSCC from patients from this region [18]. In the present study, we focused on the expression of the c-Myc protein in the oral cancers.

Investigations into the progression of pre-neoplastic lesions suggest that the c-Myc oncoprotein is expressed in the various stages, including benign keratoses [19]. A plethora of molecular changes are identified including mutations in ras as well as p53 and elevated expression of c-Myc [20]. c-Myc over-expression is also correlated with poor prognosis of OSCC [21]. Multiple gene amplifications, including c-Myc, were observed in head and neck SCC [22] and the over-expression of the cyclin-dependent Kinase 4 (Cdk4) either by gene amplification or through the c-Myc regulated pathway may also play a key role in the progression of these tumours [23].

In an investigation of oral cancers from the Eastern Indian population, of all the OSCC cases studied, 56% had anti-Myc immunoreactivity [24]. In our studies, c-Myc expression was observed in tumours from all stages, suggesting possible involvement during the various stages of the neoplastic process. This can be visualized especially in light of the fact that c-Myc is a regulator of multiple cell signalling pathways including hTERT, which is universally activated in various malignancies and is critical for maintenance of cellular immortalization. Eversole and Sapp [19] also noted c-Myc expression in both pre-cancerous (keratoses) and early stage lesions in patients from North America. In lip carcinogenesis, c-Myc expression was evident in only very late stages of the disease [25]. Hence, there is a broad spectrum of c-Myc activation in various head and neck cancers from different parts of the world.

Detection of the c-Myc protein in the majority of the OSCC we analysed attests to the seminal role, this molecule plays in the neoplastic progression. With an array of signalling pathways that can be regulated by c-Myc, it is conceivable that the oncoprotein is involved in several cascades of events necessary for initiation and maintenance of the neoplastic state at various stages. Additionally, recent studies on the management of osteosarcoma has unveiled that the c-Myc status may contribute to therapeutic response and clinical outcome [26]. Elevated expression of this oncogene exhibited unfavourable clinical outcome when methotrexate treatment was used. Methotrexate is a preferred chemotherapeutic agent in oral cancer treatment and if the aforementioned scenario also applies for OSCC, appropriate clinical strategies need to be designed based on the presence/absence of the c-Myc oncoprotein in oral cancer. Furthermore, tumours that over-express the Myc protein should be amenable to therapeutic modalities such as anti-sense and siRNA that specifically target c-Myc resulting in efficacious management of oral cancers.

## Figures and Tables

**Table 1:**
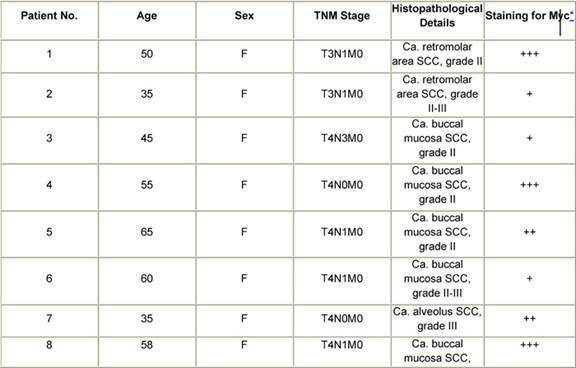

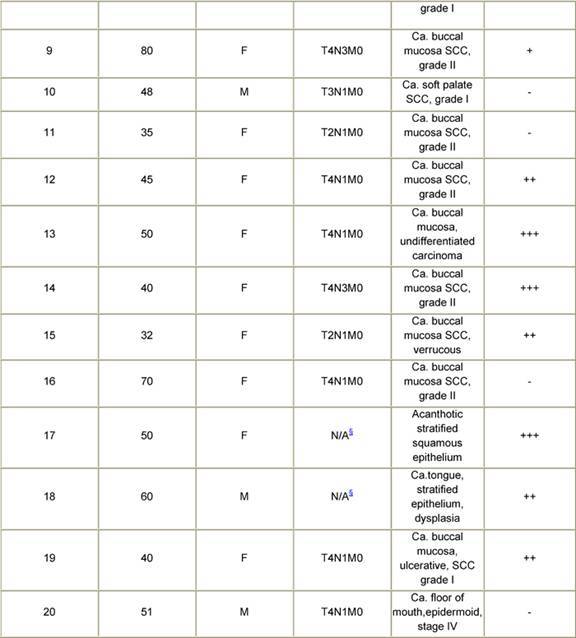

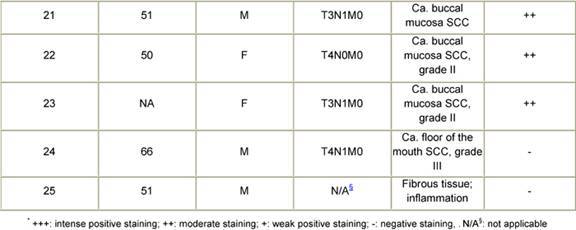
Patient history, tumour pathology and immunohistochemical staining for Myc in oral cancers

## References

[1] Gupta PC, Warnakulasuriya S (2002). Global epidemiology of areca nut usage. Addict Biol.

[2] Nair U, Bartsch H, Nair J (2004). Alert for an epidemic of oral cancer due to use of the betel quid substitutes gutkha and pan masala: a review of agents and causative mechanisms. Mutagenesis.

[3] Chen CL, Chi CW, Liu TY (2002). Hydroxyl radical formation and oxidative DNA damage induced by areca quid in vivo. J Toxicol Environ Health A.

[4] Jeng JH, Chang MC, Hahn LJ (2001). Role of areca nut in betel quid-associated chemical carcinogenesis: current awareness and future perspectives. Oral Oncol.

[5] Gangane N, Chawla S, Anshu, Gupta SS, Sharma SM (2007). Reassessment of risk factors for oral cancer. Asian Pac J Cancer Prev.

[6] Goessel G, Quante M, Hahn WC, Harada H, Heeg S, Suliman Y (2005). Creating oral squamous cancer cells: a cellular model of oral-esophageal carcinogenesis. Proc Natl Acad Sci USA.

[7] Eilers M, Eisenman RN (2008). Myc’s broad reach. Genes Dev.

[8] Saranath D, Bhoite LT, Deo MG (1993). Molecular lesions in human oral cancer: the Indian scene. Eur J Cancer B Oral Oncol.

[9] Sugerman PB, Joseph BK, Savage NW (1995). Review article: The role of oncogenes, tumour suppressor genes and growth factors in oral squamous cell carcinoma: a case of apoptosis versus proliferation. Oral Dis.

[10] Kim NW, Piatyszek MA, Prowse KR, Harley CB, West MD, Ho PL, Coviello GM, Wright WE, Weinrich SL, Shay JW (1994). Specific association of human telomerase activity with immortal cells and cancer. Science.

[11] Pai RB, Pai SB, Kukhanova M, Dutschman GE, Guo X, Cheng YC (1998). Telomerase from human leukaemia cells: properties and its interaction with deoxynucleoside analogues. Cancer Res.

[12] Dwyer J, Li H, Xu D, Liu JP (2007). Transcriptional regulation of telomerase activity: roles of the Ets transcription factor family. Ann N Y Acad Sci.

[13] Lo Muzio L (2001). A possible role for the WNT-1 pathway in oral carcinogenesis. Crit Rev Oral Biol Med.

[14] Miyashita H, Mori S, Motegi K, Fukumoto M, Uchida T (2003). Pin1 is overexpressed in oral squamous cell carcinoma and its levels correlate with cyclin D1 overexpression. Oncol Rep.

[15] Chen YJ, Lin SC, Kao T, Chang CS, Hong PS, Shieh TM, Chang KW (2004). Genome-wide profiling of oral squamous cell carcinoma. J Pathol.

[16] Pai SB, Pai RB, Johnston RN (2004). Overexpression of c-myc by amplification of negative promoter domain. J Biol Chem.

[17] Ponzielli R, Katz S, Barsyte-Lovejoy D, Penn LZ (2005). Cancer therapeutics: targeting the dark side of Myc. Eur J Cancer.

[18] Pai SB, Pai RB, Lalitha RM, Kumaraswamy SV, Lalitha N, Johnston RN, Bhargava MK (2006). Expression of oncofoetal marker carcinoembryonic antigen in oral cancers in South India—a pilot study. Int J Oral Maxillofac Surg.

[19] Eversole LR, Sapp JP (1993). c-myc oncoprotein expression in oral precancerous and early cancerous lesions. Eur J Cancer B Oral Oncol.

[20] Field JK (1992). Oncogenes and tumour-suppressor genes in squamous cell carcinoma of the head and neck. Eur J Cancer B Oral Oncol.

[21] Field JK, Spandidos DA, Stell PM, Vaughan ED, Evan GI, Moore JP (1989). Elevated expression of the c-myc oncoprotein correlates with poor prognosis in head and neck squamous cell carcinoma. Oncogene.

[22] Leonard JH, Kearsley JH, Chenevix-Trench G, Hayward NK (1991). Analysis of gene amplification in head-and-neck squamous-cell carcinoma. Int J Cancer.

[23] Mishra R, Das BR (2003). Early overexpression of Cdk4 and possible role of KRF and c-myc in chewing tobacco mediated oral cancer development. Mol Biol Rep.

[24] Baral R, Patnaik S, Das BR (1998). Co-overexpression of p53 and c-myc proteins linked with advanced stages of betel- and tobacco-related oral squamous cell carcinomas from eastern India. Eur J Oral Sci.

[25] de Rosa I, Staibano S, Lo Muzio L, Delfino M, Lucariello A, Coppola A, De Rosa G, Scully C (1999). Potentially malignant and malignant lesions of the lip. Role of silver staining nucleolar organizer regions, proliferating cell nuclear antigen, p53, and c-myc in differentiation and prognosis. J Oral Pathol Med.

[26] Scionti I, Michelacci F, Pasello M, Hattinger CM, Alberghini M, Manara MC (2008). Clinical impact of the methotrexate resistance-associated genes C-MYC and dihydrofolate reductase (DHFR) in high-grade osteosarcoma. Ann Oncol.

